# Detection of *Francisella tularensis *in ticks and identification of their genotypes using multiple-locus variable-number tandem repeat analysis

**DOI:** 10.1186/1471-2180-8-152

**Published:** 2008-09-17

**Authors:** Fang Zhang, Wei Liu, Xiao-Ming Wu, Zhong-Tao Xin, Qiu-Min Zhao, Hong Yang, Wu-Chun Cao

**Affiliations:** 1 Beijing Institute of Microbiology and Epidemiology, State Key Lab of Pathogen and Biosecurity, 20 Dong-Da-Jie Street, Fengtai District, Beijing 100071, PR China; 2 Department of Pathology & Laboratory Medicine, Emory University School of Medicine, Atlanta, GA, USA

## Abstract

**Background:**

Tularemia was reported in China over 50 years ago, however, many epidemical characteristics remain unclear. In the present study, the prevalence of *Francisella tularensis *in ticks was investigated during an epidemiological surveillance in China and then we measured their genetic diversity by conducting multiple-locus variable- number tandem repeat analysis (MLVA).

**Results:**

1670 ticks from 2 endemic areas (Inner Mongolia Autonomous Region and Heilongjiang Province) and 2 non-endemic areas (Jilin and Fujian Provinces) were collected and tested for evidence of tularemia by nested PCR. The prevalence of *Francisella tularensis *in ticks averaged 1.98%. The positive rates were significantly different among tick species, with *Dermacentor silvarum *and *Ixodes persulatus *responsible for all positive numbers. All *F. tularensis *that were detected in ticks belonged to *F. tularensis *subsp. *holarctica *and MLVA disclosed genetic diversity. One subtype was identified in 17 of 33 positive tick samples in three different study areas. Another subtype belonging to *F. tularensis *subsp. *holarctica *genotype was described for the first time in the current study.

**Conclusion:**

The study showed two tick species, *D. silvarum *and *I. persulatus *harboring the pathogen of tularemia in natural environment, indicating these two tick species might have a role in tularemia existence in China. MLVA results disclosed the genetic diversity *F. tularensis *and identified one genotype as the most prevalent among the investigated ticks in China.

## Background

Tularemia, also known as rabbit fever or deer-fly fever, is caused by the gram-negative intracellular pathogen *Francisella tularensis (F. tularensis*), which is highly infectious and considered as a potential bioweapon. Currently there are four recognized subspecies of *F. tularensis*: subsp. *tularensis*, *holarctica*, *mediasiatica *and *novicida*. The former two subspecies are clinically important. *F. tularensis *is widely distributed in the Northern hemisphere and is capable of infecting hundreds of different vertebrates and invertebrates [1-3]. In addition, a variety of arthropods, including multiple genera of ticks as well as deer flies, fleas, mites, and mice, have been found to be naturally infected or experimentally competent as vectors. In many endemic areas, ticks play an important role in transmission of the disease from animals to humans [3], with the genera *Amblyomma*, *Dermacentor*, *Haemaphysalis*, *Ixodes *and *Ornithodoros *acting as main vectors [4].

In China, *F. tularensis *was isolated in *Citellus dauricus *from Tongliao region, Inner Mongolia municipality as early as in 1957 [5,6]. The first human tularemia epidemic, including 14 reported cases, which was caused by contact with infected hares, was reported in Heilongjiang province in 1959 [7]. The natural foci of the disease were subsequently identified in Tibet and Xinjiang municipalities [8-10]. The agent was detected and isolated from patients, *Ixodex liberelis*, *Dermacentor marginatus *and wolly hares (*Lepus oiostolos*) from Qinghai Province and Tibet, Xinjiang Autonomous Regions during 1962–1986 [8-11]. An outbreak of tularemia occurred in Shandong province in 1986. All the 31 reported patients were workers in a food-processing factory, where hares were slaughtered and processed [12]. Serological investigations demonstrated that the seropositive reaction to *F. tularensis *was significantly associated with tick exposure [8,13]. However, little is known about the prevalence of *F. tularensis *in ticks in China, lack of such information has inhibited our fully understanding of the disease ecology and also under-estimated the threat of the pathogen to human health where ticks are abundant. In the present study, we try to investigate the presence of *F*. *tularensis *in ticks, and then to identify their genotypes using multiple-locus variable-number tandem repeat analysis (MLVA).

## Results

### Prevalence of *F. tularensis *in ticks

Out of 1670 adult ticks examined, thirty three were positive for the *fopA *gene of *F. tularensis*. The overall positive rate was 1.98%. All positive samples amplified the 220-bp fragment for the *ppI*-*helicase *gene that identifies *F. tularensis *subsp.*holarctica *(GenBank accession no. EU526911). Thus, all ticks in the present study were infected with *F. tularensis *subsp.*holarctica*. The positive rates of *F. tularensis *in *D. silvarum *were 3.67% and 2.33% in *I. persulatus *(Table 1), while all of the 144 *Haemaphysalis verticalis *and 299 *Boophilus microplus *were PCR-negative. The difference in positive rates among different species was significant (χ^2 ^= 14.366, *P *= 0.002). We were able to detect *F. tularensis *in ticks from Jilin, Heilongjiang and Inner Mongolia, but not from Fujian during the present study. The positive rates varied according to their geographical origins (Table 1), with minor statistical significance (χ^2 ^= 7.865, *P *= 0.049).

**Table 1:** Detection for *F. tularensis *in ticks by species and geographic origins in China

Tick species	No. positive/no. detected (%)				
	
	Jilin	Heilongjiang	Inner Mongolia	Fujian	Total
*Dermacentor sylvarum*	12/327(3.67)	---*	---	---	12/327(3.67)
*Ixodes persulatus*	0/100 (0)	9/400(2.25)	12/400 (3.00)	---	21/900(2.33)
*Haemaphysalis verticalis*	---	---	0/144(0)	---	0/144(0)
*Boophilus microplus*	---	---	---	0/299(0)	0/299(0)

Total	12/427(2.81)	9/400(2.25)	12/544 (2.21)	0/299(0)	33/1670(1.98)

---, No ticks captured

### MLVA

Two *F. tularensis *MLVA markers (Ft-M3 and Ft-M10) were successfully amplified from the 33 samples that tested positive for both the *fopA *gene and *PPI-helicase *PCRs. The sequences from Ft-M10 were invariant, 2 copies and 361 bp in size (GenBank accession no. EU544140), same as previously reported [14]. The Ft-M3 loci had two allelic variants (9A: AACAAAGAC and 9E: AATAAGGAT), exactly as defined by Johansson for *F. tularensis *subsp. *holarctica *[15,16]. The sequence size from Ft-M3 ranged greatly from 270 to 369 bp and the repeating units differed from 7 to 18 copies (GenBank accession no. EU544141 to EU544147). Based on the copy numbers of two loci, seven genotypes were identified (Table 2). The Ft-M3 genotype containing 9 repeat copies was detected at 3 study sites in over half of the *F. tularensis *positive ticks (17/33, 51.5%). The significance of this genotype-host association remains unknown. Different genotypes could be detected from the same location (Table 2), among which, one Ft-M3 genotype containing 7 repeat copies from *I. persulcatus *was unique which had been found existence in *F. tularensis *subsp. *tularesnsis *in Goethert's study [17].

**Table 2:** Genotypes of *F. tularensis *detected from ticks in China

Geographical sites	Number	Hosts	Genotypes for two marker	
			
			Ft-M3	Ft-M10
Heilongjiang	1	*Ixodes persulcatus*	7	2
	2	*I. persulcatus*	8	2
	6	*I. persulcatus*	9	2
Jilin	6	*Dermacentor sivarum*	9	2
	2	*D.sivarum*	15	2
	4	*D.sivarum*	16	2
Inner Mongolia	5	*Ixodes persulcatus*	9	2
	3	*I. persulcatus*	15	2
	3	*I. persulcatus*	14	2
	1	*I. persulcatus*	18	2

## Discussion

Although *F. tularensis *can be cultured in the laboratory, it requires enriched growth media and BSL3 facilities for isolation, these problems, together with the difficulty of recognizing the bacterium make it lack rapid and safe methods for laboratory identification [18]. Nowadays, various PCR approaches amplifying different genomic segments have been applied to detecting *F. tularensis *[19-22]. The evaluations were performed during epidemics in Sweden in 1995 and 1998, respectively. It proved PCR detection of *F. tularensis *was more sensitive than culture [18]. In present study, nested-PCR method which targeting *fopA *gene of *F. tualrensis *[17,23] was used to detect *F. tularensis *in ticks from four provinces of China. We were able to detect *F. tualrensis *in *D. silvarum *and *I. persulcatus *from three northern provinces of China. The prevalence in ticks was 1.98%, which is comparable with the prevalence of 2.1%–2.8% in *D. reticulates *from European countries [4,24]. Actually, as early as 2004, during an epidemical survey we have detected *F. tularensis *in rodents from these three provinces [25]. The present findings, combined with our previous detection result in rodents, adds evidence to our belief that tularemia may be only confined to the northern part of China. During our study, we were able to detect *F. tularensis *in *D. silvarum *and *I. persulatus*, whereas not from *Haemaphysalis verticalis *and *Boophilus microplus*. Moreover, the *F. tularensis *strains in China were mainly isolated from these two tick genera [8-10]. The principal tick vectors seemed only include the genus *Dermacentor *and *Ixodes *in China, because we have found no evidence that other tick genera was involved in *F. tularensis *to date.

For the first time, our study identified the infection of *F. tularensis *in ticks from Jilin Province. Actually, natural infection of *F. tularensis *in rodents had been recorded from same study sites in 2006 [25]. Geographically, Jilin Province is adjacent to Heilongjiang Province where first human tularemia cases were reported and natural focus was established as early as in 1958 [7]. The study sites from two provinces were close to each other and they have the same ecological environment. So it was not surprising that we were able to detect *F. tularensis *in ticks from Jilin Province. However, it is still too early to state that tularemia natural focus exists in Jilin Province because until now we have not obtained the isolate and there is no any case report.

Tandem repeat regions are known to be highly variable and successfully used for typing bacterial species, even for discriminating individual isolates [15,17,26-28]. During our study, two loci (Ft-M3 and Ft-M10) were used to measure the genetic diversity of *F. tularensis *detected in the study. The sequence of the 16-bp repeating unit of Ft-M10 was invariant, this result was consistent with our previous study results [14]. Perhaps there was only one variant from Ft-M10 locus among Chinese *F. tularensis*, although about 10 variants had been identified [30], among which 3 variants for *F. tularensis *subsp. *holarctica *were identified [15]. 7 variants were identified for 33 positive samples from Ft-M3 locus, among which one unique type containing 7 repeat copies was firstly identified in *F. tularensis *subsp. *holarctica*, this type had been identified from *F. tularensis *subsp. *tularensis *infected tick samples in Goethert's study [17]. According to the sequences, we are sure that the *F. tularensis *detected in present study can all be grouped as the *F. tularensis *subsp. *holarctica *(type B), not *F. tularensis *subsp. *tularensis*. The type containing 9 repeat copies from Ft-M3 locus seemed to be dominant in the detected ticks, actually in Goethert's study they had identified one genotype (also from Ft-M3 locus containing 10 repeat copies) only existing in ticks, which belonged to *F. tularensis *subsp.*tularensis *(type A). A variety of samples should be included to illuminate whether there are differences in various genotype among host ranges.

## Conclusion

This study showed the role of two tick species in harboring the pathogen of tularemia in natural environment in China. In endemic foci where these genera ticks are abundant, public health officials and clinicians should be alert to the presence of tularemia, although it is not notifiable disease in China. In addition, MLVA results disclosed the genetic diversity of detected *F. tularensis *and identified one dominant genotype existing in ticks. Future work will involve more markers other than Ft-M3 and Ft-M10 detected in multiple-labs so to make comparisons with isolates of worldwide origin possible [29,30].

## Methods

### Tick collection

Adult ticks were collected from Inner Mongolia Autonomous Region, Heilongjiang Province, Jilin Province and Fujian Province. Ticks from Inner Mongolia Autonomous Region were collected in April 2005. Others were collected during April to June in 2006. The first three collection sites are forested highlands in northeastern China, belonging to Great Xing'an Mountains and Small Xing'an Mountains with an elevation 500–825 m. There are many marsh existing in above three regions. The main vegetation types are coniferophyte, and in marsh region, Tansy, reeds, and Artemisia are dominant. The temperature ranges from -40°C to 30°C. The study site in Fujian Province is wooded foothills in southern China. The main vegetation is broad-leaved plant. At least 5 different sites were sampled for each province. Ticks were collected by dragging a cloth over the vegetation and were stored alive until brought into laboratory. In the laboratory, ticks were examined morphologically and sorted by species, developmental stage under microscope. After species identification, tick specimens were stored at -20°C for further DNA extraction.

### DNA extraction

Ticks were processed individually. DNA extraction was performed by a modification of a previously described method [31]. Briefly, each tick was soaked in 70% ethanol for 10 minutes, and then rinsed three times in sterile water. Then the tick was placed into microtube and mechanically disrupted with sterile scissors in 50 μl of DNA extraction buffer (10 mM Tris, pH 8.0, 2 mM EDTA, 0.1% SDS, 500 μg of proteinase K/ml). Each sample was incubated for 2 h at 56°C and then boiled at 100°C for 10 min to inactivate proteinase K. After centrifugation, the supernatant was transferred to a fresh sterile microtubule and purified by extracting twice with an equal volume of phenol-chloroform before use. Then TE (10 mM Tris, pH 8.0, 2 mM EDTA) was added to dissolve DNA for PCR use.

### PCR amplification

Samples were screened for evidence of *F. tularensis *by nested PCR targeting the *fopA *gene, as described previously [17,23]. All positive specimens for the *fopA *gene were re-tested to obtain subspecies confirmation by amplifying the *ppI*-helicase region of *F*. *tularensis *gene structure using the primer pair of C6 (AGGCGGAGATCTAGGAACCTTT, GenBank accession no. AF247642) and C8 (AGCCCAAGCTGACTAAAATCTTT, GenBank accession no. AF247685). This PCR yielded a variable-length amplicon due to a 30-bp deletion in *F. tularensis *subsp.*holarctica*: for *F. tularensis *subsp.*holarctica*, the amplicon was 220 bp (GenBank accession no. EU526911), and for *F. tularensis *subsp.*tularensis*, the amplicon was 250 bp in length [15]. All the PCR products were separated by 3% agarose gel electrophoresis, stained with ethidium bromide, and visualized under UV light. Negative controls and positive controls (Chinese first *F. tularensis *strain isolated from *Citellus dauricus *in 1957 from Inner Mongolia) were applied to ensure validation of the amplification. To minimize contamination, DNA extraction, the reagent setup, amplification, and agarose gel electrophoresis were performed in separate rooms. Amplicons from all PCR assays were sequenced to verify the validity of the PCR. For sequencing, amplicons were excised from the gels, purified through spin columns (Qiagen, Hilden, Germany) and sequenced on automated DNA sequencer (ABI PRISM 377, Perkin-Elmer, Foster City, USA). All sequencing primers were amplification primers which were read for both strands.

### Multiple-locus variable number tandem repeat analysis (MLVA)

Using MLVA, two markers, Ft-M3 and Ft-M10 (previous designations SSTR9 and SSTR16, respectively) were used to identify the genetic diversity among positive ticks as previously described [14,15]. All amplicons obtained from two markers were sequenced on an automated DNA sequencer (ABI PRISM 377 Parkin-Elmer, Inc). The number of repeated units for each sample was counted. The genotype was defined simply as the number of repeats for Ft-M3 followed by that for Ft-M10.

### Nucleotide sequence accession numbers

Nucleic acid sequences representative of each genotype and sequence from subspecies confirmation were deposited in GenBank under accession numbers EU526911, EU544140 to EU544147.

## Authors' contributions

FZ and WL carried out the samples detection and MLVA analysis, participated in ticks sampling and drafted the manuscript, ZTX performed the statistical analysis and drafted the manuscript. HY, XMW and QMZ participated in ticks sampling. WCC conceived of the study, participated in its design, coordinated the field investigation and drafted the manuscript. All authors read and approved the final manuscript.
